# Supplementary material for: Aubergine and piRNAs promote germline stem cell self‐renewal by repressing the proto‐oncogene Cbl
Source: EMBO J. 2017 Oct 13;36(21):3194–211. doi: 10.15252/embj.201797259 (PMC5666619; doi:10.15252/embj.201797259)
Supplement: Supplementary file 1 — Appendix [file EMBJ-36-3194-s001.pdf]

Aubergine and piRNAs repress the proto-oncogene *Cbl* for germline stem cell self-renewal

Patricia Rojas-Ríos[1], Aymeric Chartier[1], Stéphanie Pierson[1] and Martine Simonelig[1#]

Appendix

Table of Contents:

- Appendix Figure S1, p. 2
- Appendix Figure S2, p. 3
- Appendix Figure S3, p. 4
- Appendix References, p. 5

A

Gene	Known function in stem cell lineages	Aub iCLIP in embryos (Barckmann et al., 2015)	Up-regulated in *aub* GSCs
nanos	Ref. 1 and 2	YES	+
brat	Ref. 3	NO	-
mei-P26	Ref. 4	NO	+
fused	Ref. 5	NO	+
pgc	Ref. 6	YES	-
aret	Ref. 7	YES	-
lola	Ref. 8	YES	-
Cbl	Ref. 9	YES	+

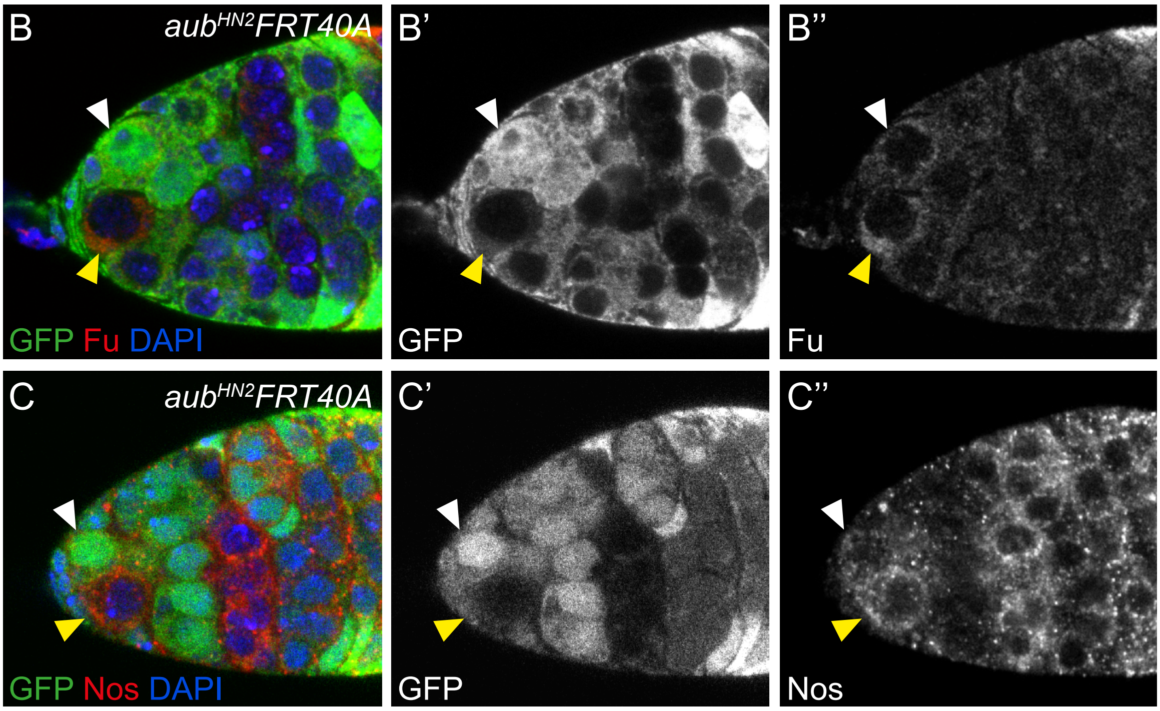

Appendix Figure S1: Aub candidate target mRNAs
(A) List of candidate target mRNAs of Aub in GSCs. Increase of protein levels in aub^{HN2} mutant versus control ($aub^{HN2/+}$) GSCs was determined using the clonal analysis, as shown in (B-C''). +: increased protein levels; -: no increase. Ref. 1 (Wang & Lin, 2004); Ref. 2 (Gilboa & Lehmann, 2004); Ref. 3 (Harris et al, 2011); Ref.4 (Neumuller et al, 2008); Ref. 5 (Xia et al, 2010); Ref. 6 (Hanyu-Nakamura et al, 2008); Ref. 7 (Xin et al, 2013); Ref. 8 (Davies et al, 2013); Ref. 9 (Sanada et al, 2009).
(B-C'') Immunostaining of aub^{HN2} mosaic germaria 7 days after clone induction, with anti-GFP (green) and anti-Fused (Fu, B, B'') or anti-Nanos (Nos, C, C'') (red). DAPI (blue) was used to visualize DNA. White arrowheads indicate control ($aub^{HN2/+}$) GSCs; yellow arrowheads indicate aub^{HN2} mutant GSCs.

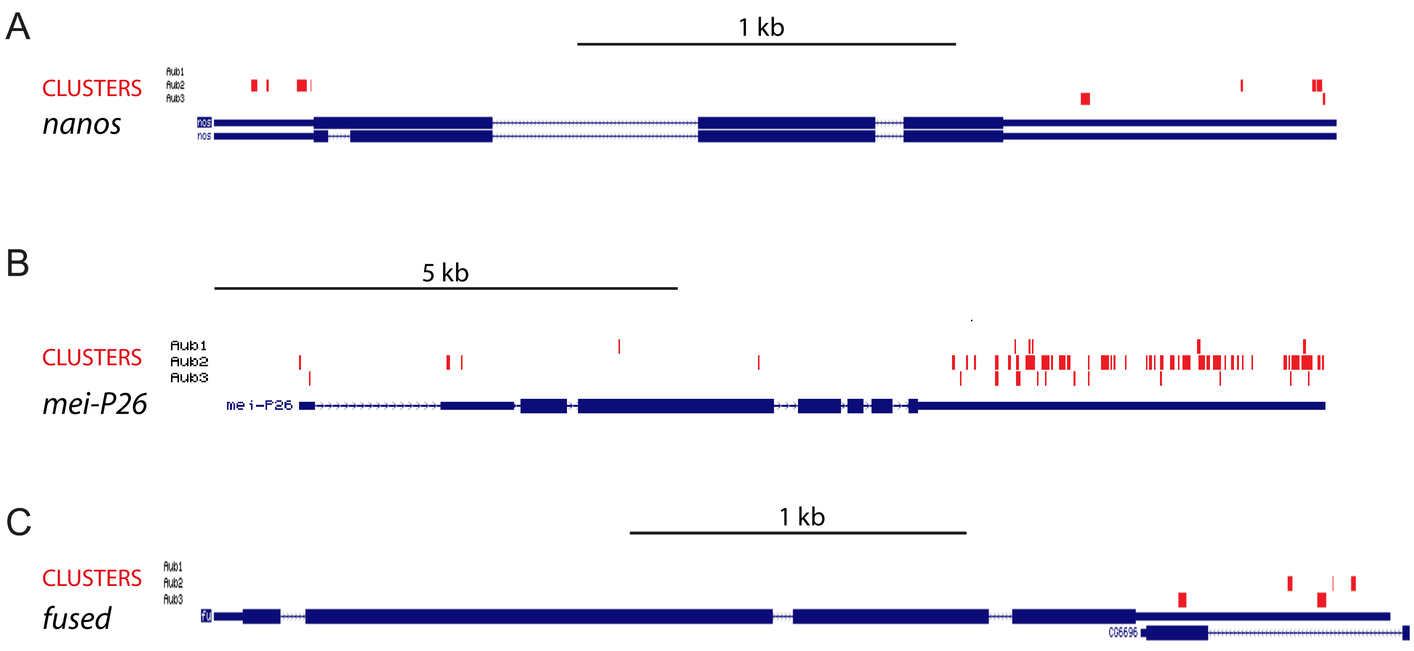

Appendix Figure S2: GFP-Aub iCLIP in GSCs *in nanos, mei-P26* and *fused* mRNAs
(A-C) Significant GFP-Aub crosslinks in cultured GSCs (Ma et al, 2017), in *nanos* (A), *mei-P26* (B) and *fused* (C) mRNAs. Thin boxes are 5'- and 3'UTRs, lines are introns, and thick boxes are exons. Significant crosslink clusters are indicated in red.

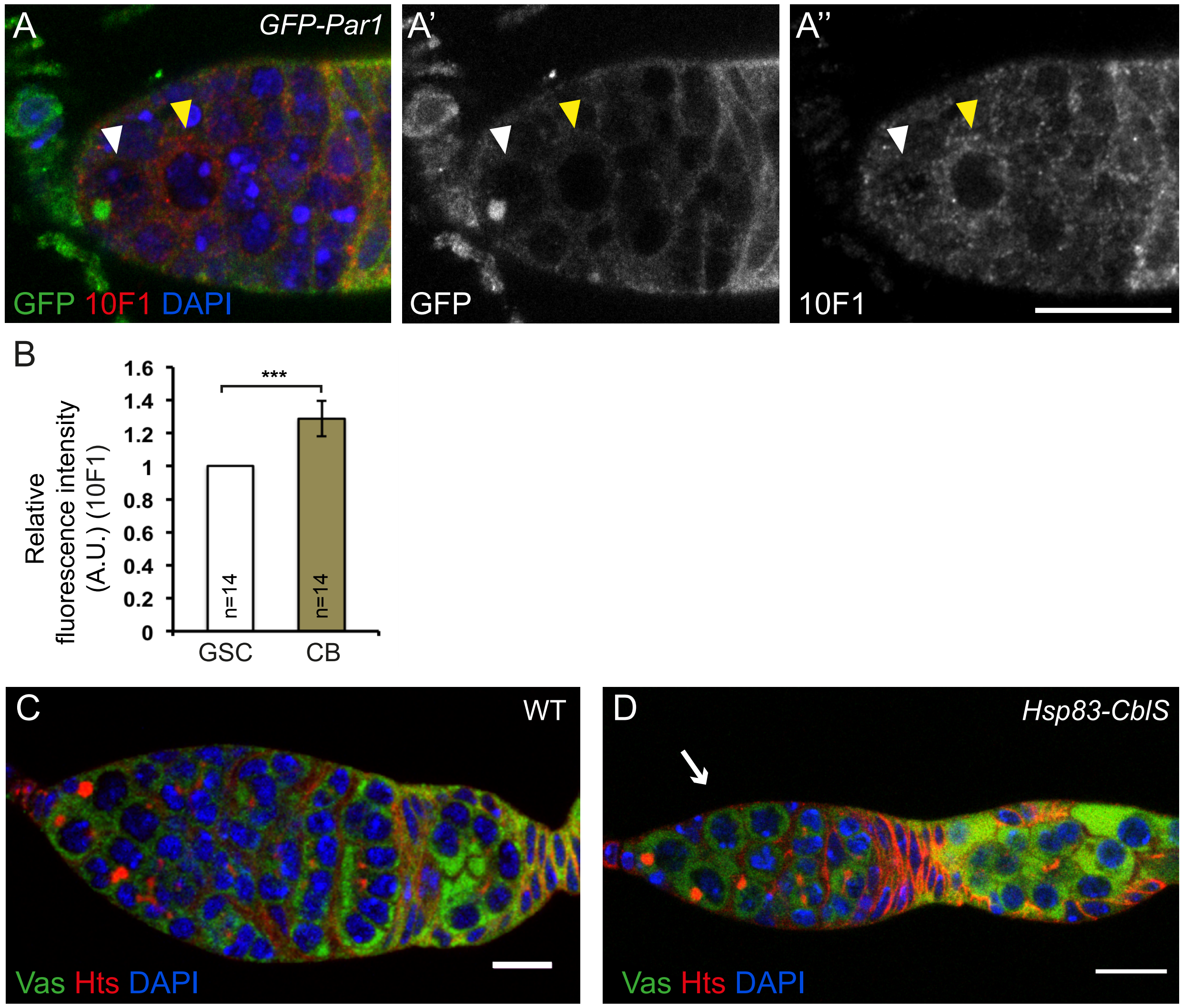

Appendix Figure S3: Cbl expression pattern in the germarium

(A-A'') Immunostaining of germaria expressing *GFP-Par1* to label spectrosomes with anti-GFP (green) and anti-Cbl 10F1 (red). DAPI (blue) was used to visualize DNA. White arrowheads indicate GSCs; yellow arrowheads indicate cystoblasts.

(B) Quantification of Cbl protein levels in GSCs and cystoblasts using fluorescence intensity of immunostaining with 10F1 antibody. Fluorescence intensity was measured in arbitrary units using ImageJ. The number of scored cells (n) is indicated. Intensity in GSCs was set to 1. The error bar indicates standard deviation. ****p*-value <0.001 using the two-tailed Student's *t* test.

(C, D) Immunostaining with anti-Vasa (green) and anti-Hts (red) of wild-type germaria (C) and of germaria overexpressing *Cbl* with *Hsp83-CblS* (D). DNA (blue) was revealed with DAPI. The white arrow indicates the lower number of germ cells. Scale bar: 10 µm in A, C, D.

References

Davies EL, Lim JG, Joo WJ, Tam CH, Fuller MT (2013) The transcriptional regulator lola is required for stem cell maintenance and germ cell differentiation in the Drosophila testis. *Developmental biology* **373:** 310-321

Gilboa L, Lehmann R (2004) Repression of primordial germ cell differentiation parallels germ line stem cell maintenance. *Current biology* **14:** 981-986

Hanyu-Nakamura K, Sonobe-Nojima H, Tanigawa A, Lasko P, Nakamura A (2008) Drosophila Pgc protein inhibits P-TEFb recruitment to chromatin in primordial germ cells. *Nature* **451:** 730-733

Harris RE, Pargett M, Sutcliffe C, Umulis D, Ashe HL (2011) Brat promotes stem cell differentiation via control of a bistable switch that restricts BMP signaling. *Developmental cell* **20:** 72-83

Ma X, Zhu X, Han Y, Story B, Do T, Song X, Wang S, Zhang Y, Blanchette M, Gogol M, Hall K, Peak A, Anoja P, Xie T (2017) Aubergine Controls Germline Stem Cell Self-Renewal and Progeny Differentiation via Distinct Mechanisms. *Developmental cell* **41:** 157-169 e155

Neumuller RA, Betschinger J, Fischer A, Bushati N, Poernbacher I, Mechtler K, Cohen SM, Knoblich JA (2008) Mei-P26 regulates microRNAs and cell growth in the Drosophila ovarian stem cell lineage. *Nature* **454:** 241-245

Sanada M, Suzuki T, Shih LY, Otsu M, Kato M, Yamazaki S, Tamura A, Honda H, Sakata-Yanagimoto M, Kumano K, Oda H, Yamagata T, Takita J, Gotoh N, Nakazaki K, Kawamata N, Onodera M, Nobuyoshi M, Hayashi Y, Harada H, Kurokawa M, Chiba S, Mori H, Ozawa K, Omine M, Hirai H, Nakauchi H, Koeffler HP, Ogawa S (2009) Gain-of-function of mutated C-CBL tumour suppressor in myeloid neoplasms. *Nature* **460:** 904-908

Wang Z, Lin H (2004) Nanos maintains germline stem cell self-renewal by preventing differentiation. *Science* **303:** 2016-2019

Xia L, Jia S, Huang S, Wang H, Zhu Y, Mu Y, Kan L, Zheng W, Wu D, Li X, Sun Q, Meng A, Chen D (2010) The Fused/Smurf complex controls the fate of Drosophila germline stem cells by generating a gradient BMP response. *Cell* **143:** 978-990

Xin T, Xuan T, Tan J, Li M, Zhao G, Li M (2013) The Drosophila putative histone acetyltransferase Enok maintains female germline stem cells through regulating Bruno and the niche. *Developmental biology* **384:** 1-12
