# Supplementary material for: Aubergine and piRNAs promote germline stem cell self‐renewal by repressing the proto‐oncogene Cbl
Source: EMBO J. 2017 Oct 13;36(21):3194–211. doi: 10.15252/embj.201797259 (PMC5666619; doi:10.15252/embj.201797259)
Supplement: Supplementary file 2 — Expanded View Figures PDF [file EMBJ-36-3194-s002.pdf]

Expanded View Figures

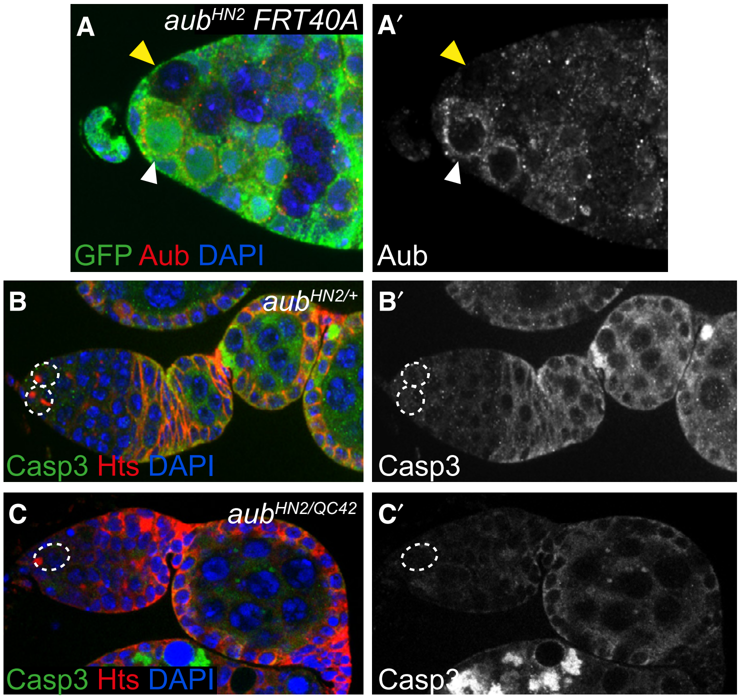

D

Percentage of germaria with Cleaved-Casp3-positive GSCs			
Genotype	7 days	14 days	21 days
aub$^{HN2/+}$	3.1% (n=65)	2% (n=98)	2% (n=51)
aub$^{HN2/QC42}$	2.6% (n=77)	1.6% (n=61)	0% (n=79)

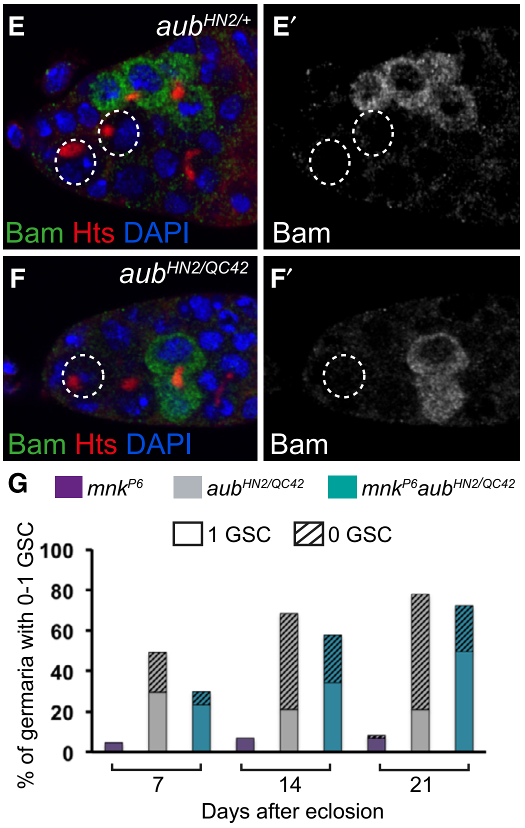

Figure EV1. GSC loss in *aub* mutant is independent of apoptosis and of Bam expression.

A, A′ *aub*^{HN2} clonal GSCs do not express Aub protein. Immunostaining of mosaic germaria with anti-GFP (green) and anti-Aub (red) 7 days after clonal induction. DAPI (blue) was used to visualize DNA. The white and yellow arrowheads indicate the control *aub*$^{HN2/+}$ and the *aub*^{HN2} clonal GSCs, respectively.

B–C′ *aub* mutant GSCs do not die by apoptosis. Immunostaining of control *aub*$^{HN2/+}$ (B, B′) and mutant *aub*$^{HN2/QC42}$ (C, C′) germaria with anti-cleaved Caspase3 (green) and anti-Hts (red). DAPI (blue) was used to visualize DNA. GSCs are outlined.

D Quantification of germaria shown in (B–C′) with cleaved Caspase3-positive GSCs in 7-, 14- and 21-day-old females. The percentages are not statistically different using the χ^2 test.

E–F′ *aub* mutant GSCs do not express Bam. Immunostaining of control *aub*$^{HN2/+}$ (E, E′) and mutant *aub*$^{HN2/QC42}$ (F, F′) germaria with anti-Bam (green) and anti-Hts (red). DAPI (blue) was used to visualize DNA. GSCs are outlined.

G Quantification of mutant germaria with 0–1 GSC shown in Fig 2A and B, showing that the GSC loss remains high in *chk2 aub* double mutant. The genotypes are indicated at the top. The number of scored germaria (*n*) is indicated in Fig 2C.

© 2017 The Authors

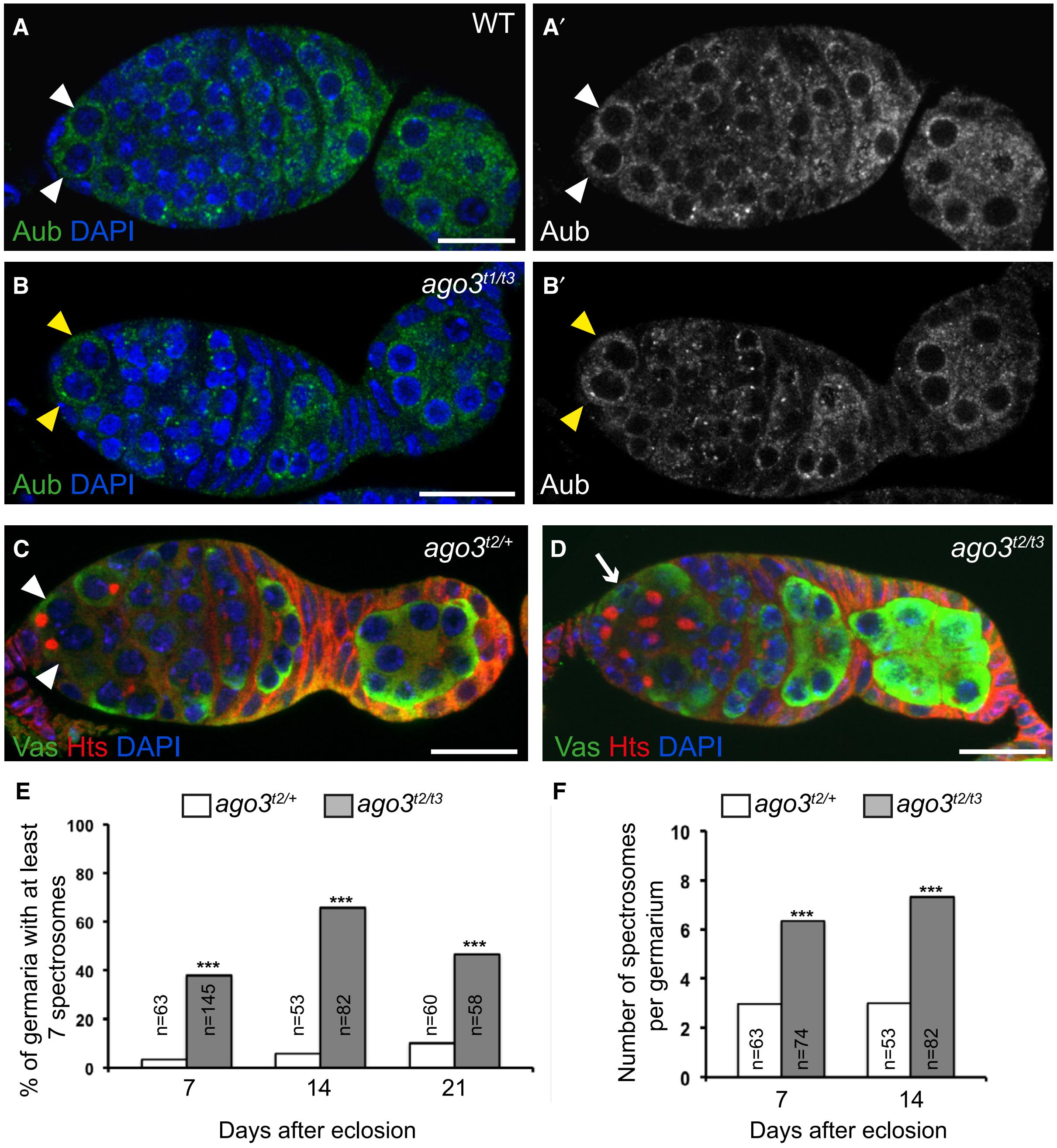

Figure EV2. GSC tumor phenotype in *ago3* mutant germaria.

A–B' Immunostaining of wild-type (A, A') and *ago3*$^{t1/t3}$ mutant (B, B') germaria with anti-Aub (green) showing that Aub expression is not affected in *ago3* mutant GSCs. DAPI (blue) was used to visualize DNA. White and yellow arrowheads indicate wild-type and mutant GSCs, respectively.

C, D Immunostaining of control *ago3*$^{t2/+}$ (C) and *ago3*$^{t2/t3}$ mutant (D) germaria with anti-Vasa (green) and anti-Hts (red). DAPI (blue) was used to visualize DNA. White arrowheads indicate GSCs, and the white arrow indicates the increased number of cells with spectrosomes.

E, F Quantification of germaria shown in (C, D) with increased number of spectrosomes (E) and quantification of spectrosomes per germarium (F). The number of scored germaria (*n*) is indicated. ****P*-value < 0.001 using the χ^2 test in (E) and the two-tailed Student's *t*-test in (F).

Data information: Scale bars: 10 µm in (A–D).

 © 2017 The Authors

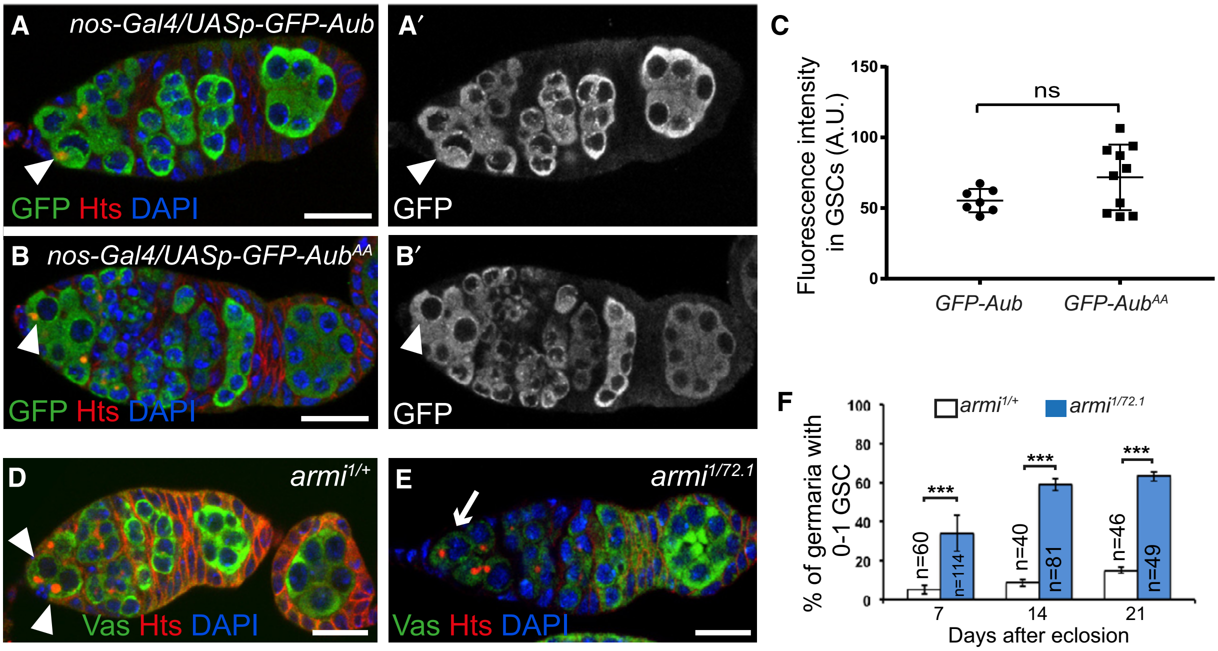

Figure EV3. Expression of GFP-Aub transgenes in GSCs, and GSC loss phenotype in *armi* mutant.

A–B′ GFP-Aub and GFP-Aub^{AA} are expressed at similar levels in GSCs using the *nos*-Gal4 driver. Immunostaining of *nos-Gal4/UASp-GFP-Aub* (A, A′) and *nos-Gal4/UASp-GFP-Aub^{AA}* (B, B′) ovaries with anti-GFP (green) and anti-Hts (red). DAPI (blue) was used to visualize DNA. White arrowheads indicate GSCs.

C Quantification of GFP-Aub and GFP-Aub^{AA} protein levels in GSCs using fluorescence intensity of immunostaining with anti-GFP shown in (A′, B′). Fluorescence intensity was measured in arbitrary units using the ImageJ software. Horizontal bars correspond to the mean and standard deviation. ns, non-significant using the two-tailed Student's *t*-test. The number of cells analyzed is shown in the figure as dots or squares.

D, E GSC self-renewal defect in *armi* mutant. Immunostaining of control *armi$^{1/+}$* (D) and *armi$^{1/72.1}$* mutant (E) germaria with anti-Vasa (green) and anti-Hts (red). DAPI (blue) was used to visualize DNA. White arrowheads indicate GSCs; the white arrow indicates reduced number of GSCs.

F Quantification of germaria with 0–1 GSC shown in (D, E), in 7-, 14- and 21-day-old females. The number of scored germaria (*n*) is indicated. Error bars represent standard deviation. ***P-value < 0.001 using the χ^2 test.

Data information: Scale bars: 10 μm in (A–B′), (D) and (E).

Figure EV4. Cbl protein levels in *ago3*, *twin*, and *armi* mutant GSCs.

A Quantification by RT–qPCR of *CblL* mRNA in dissected germaria/early egg chambers from wild-type and *aub$^{HN2/QC42}$* females. Normalization was with *RpL32* mRNA. Mean of three biological replicates. Error bars represent standard error to the mean. ns, non-significant using the two-tailed Student's *t*-test.

B ePAT assay of *mei-P26* mRNA showing elongated poly(A) tail in *twin* mutant. Ovaries from 1-day-old (germarium to stage 8) wild-type, *aub* and *twin* mutant females were used.

C–E′ Immunostaining of wild-type (C, C′), *ago3* mutant (D, D′), and *twin* mutant (E, E′) germaria with anti-Cbl 8C4 antibody (red). DAPI (blue) was used to visualize DNA. White and yellow arrowheads indicate wild-type and mutant GSCs, respectively.

F–F″ Immunostaining of *twin^{DG24102}* mosaic germaria 7 days after clone induction, with anti-GFP (green) and 8C4 anti-Cbl antibody (red). DAPI (blue) was used to visualize DNA. White arrowheads indicate control (*twin$^{DG24102/+}$*) GSCs; yellow arrowheads indicate *twin^{DG24102}* mutant GSCs.

G–H′ Immunostaining of control *armi$^{1/+}$* (G, G′) and *armi$^{1/72.1}$* mutant (H, H′) germaria with anti-Cbl 8C4 antibody (green). DAPI (blue) was used to visualize DNA. White and yellow arrowheads indicate wild-type and mutant GSCs, respectively.

I–L Quantification of Cbl protein levels in GSCs using fluorescence intensity of immunostaining with 8C4 antibody. Fluorescence intensity was measured in arbitrary units using ImageJ. Horizontal bars correspond to the mean and standard deviation. ***P-value < 0.001, *P-value < 0.05, ns, non-significant, using the two-tailed Student's *t*-test. The number of cells analyzed is shown in the figure as dots or squares (I, J).

Data information: Scale bars: 10 μm in all panels.

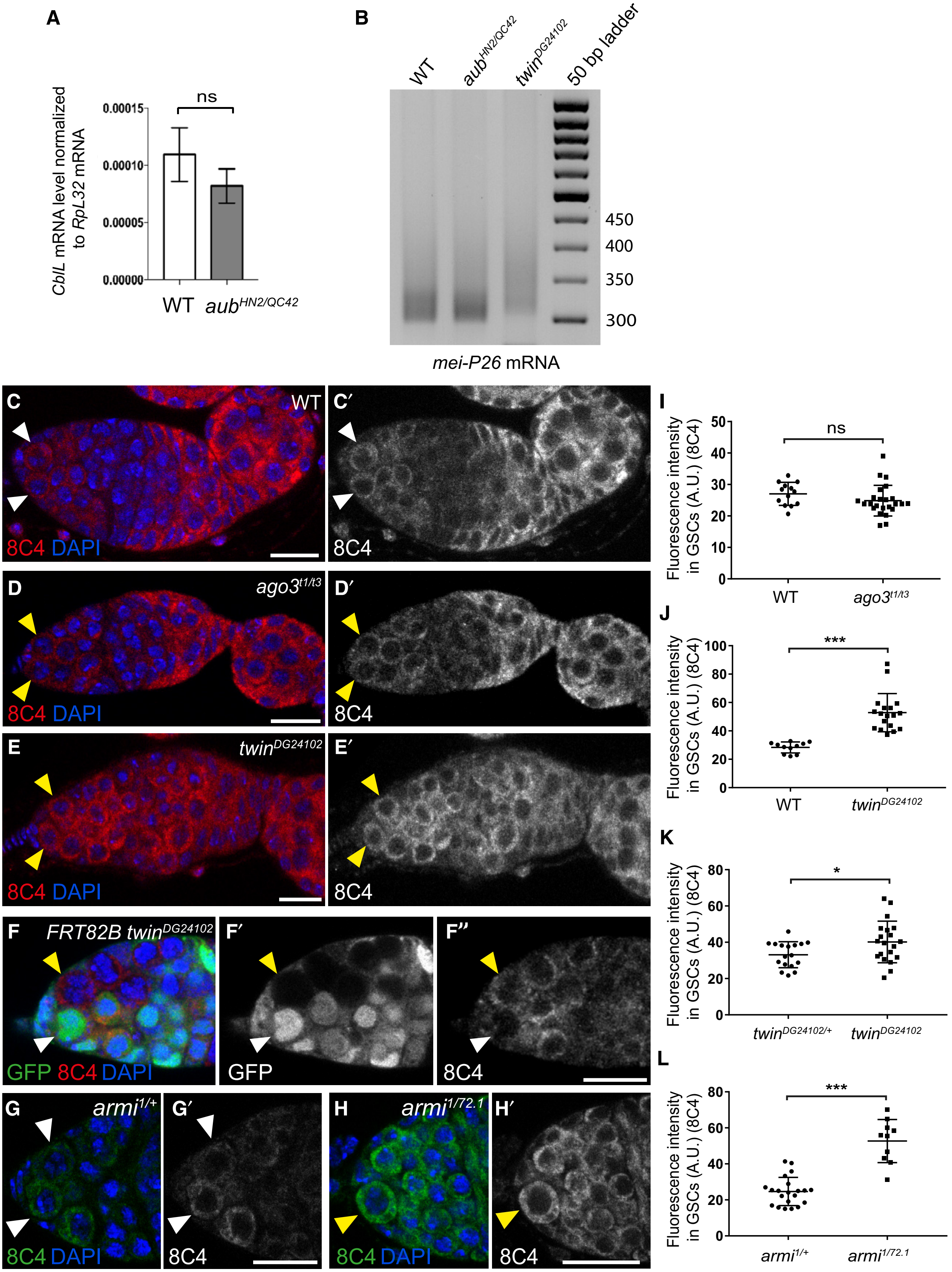

Figure EV4.

 © 2017 The Authors
