# Supplementary figures and images for: Aubergine and piRNAs promote germline stem cell self‐renewal by repressing the proto‐oncogene Cbl
Source: EMBO J. 2017 Oct 13;36(21):3194–211. doi: 10.15252/embj.201797259 (PMC5666619; doi:10.15252/embj.201797259)

WB: anti-NOT1

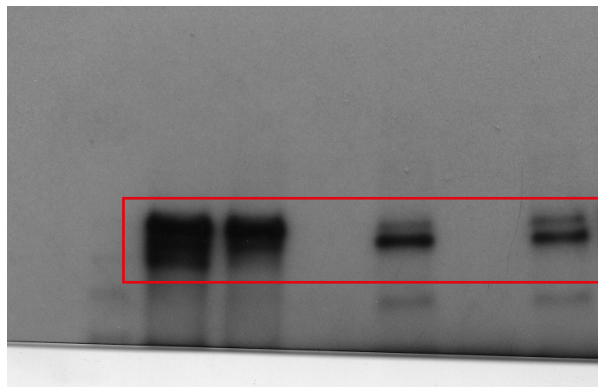

WB: anti-NOT3

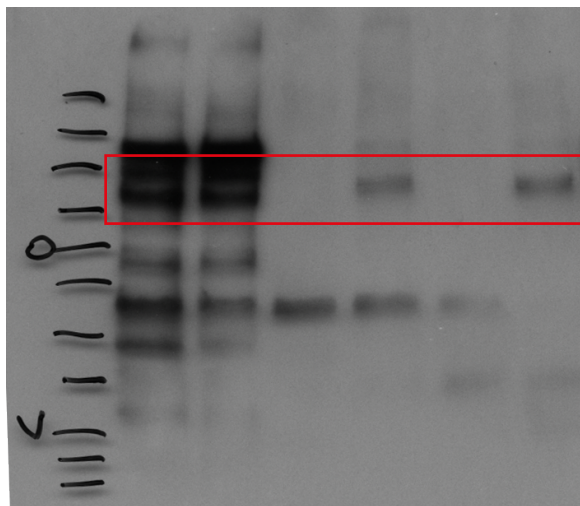

WB: anti-CCR4

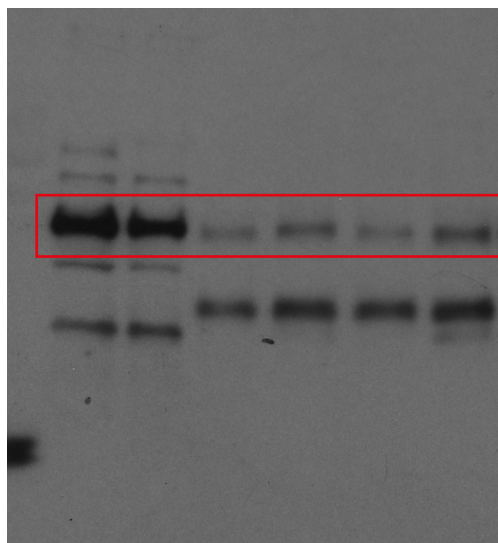

WB: anti-GFP

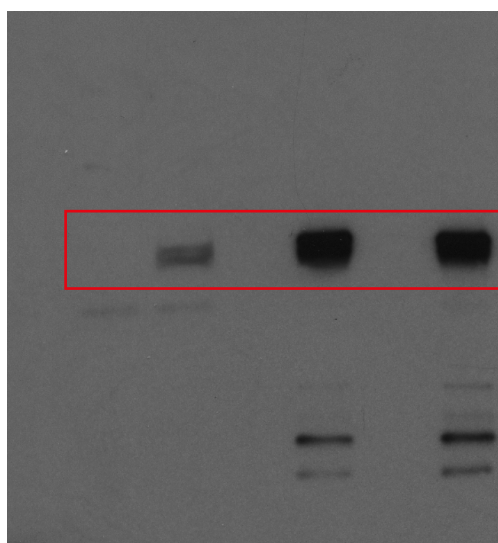

Source Data For Figure 3C

Supplement: Supplementary file 4 — Source Data for Figure 3C and D [file EMBJ-36-3194-s003.zip › EMBOJ_97259_Sourcedata_fig3C.pdf]

WB: anti-GFP

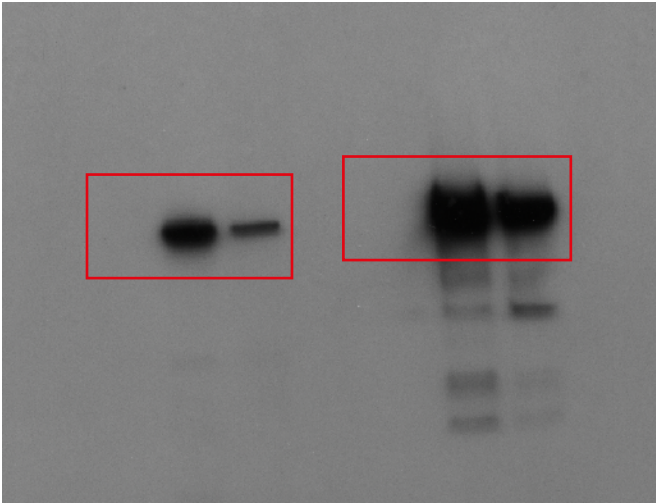

WB: anti-NOT1

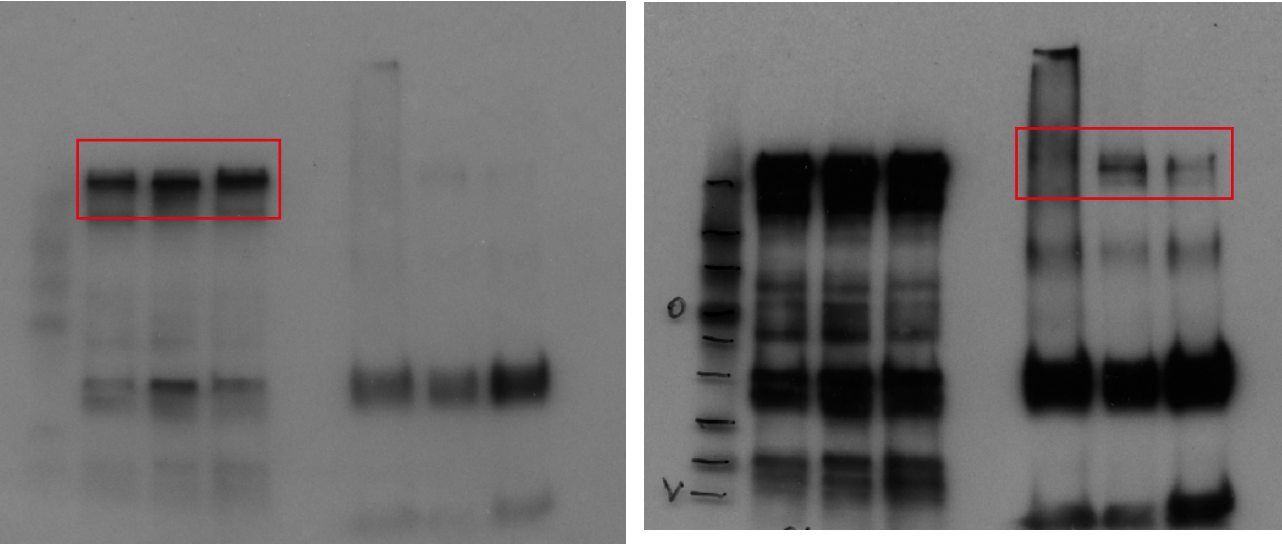

WB: anti-NOT3

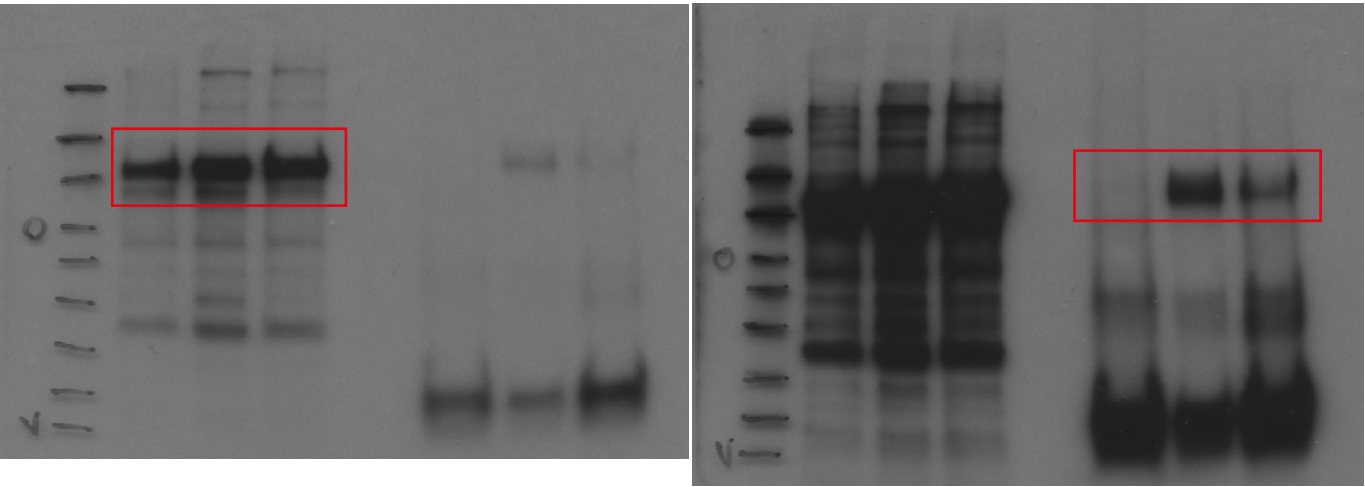

Source Data For Figure 3D

Supplement: Supplementary file 4 — Source Data for Figure 3C and D [file EMBJ-36-3194-s003.zip › EMBOJ_97259_Sourcedata_fig3D.pdf]

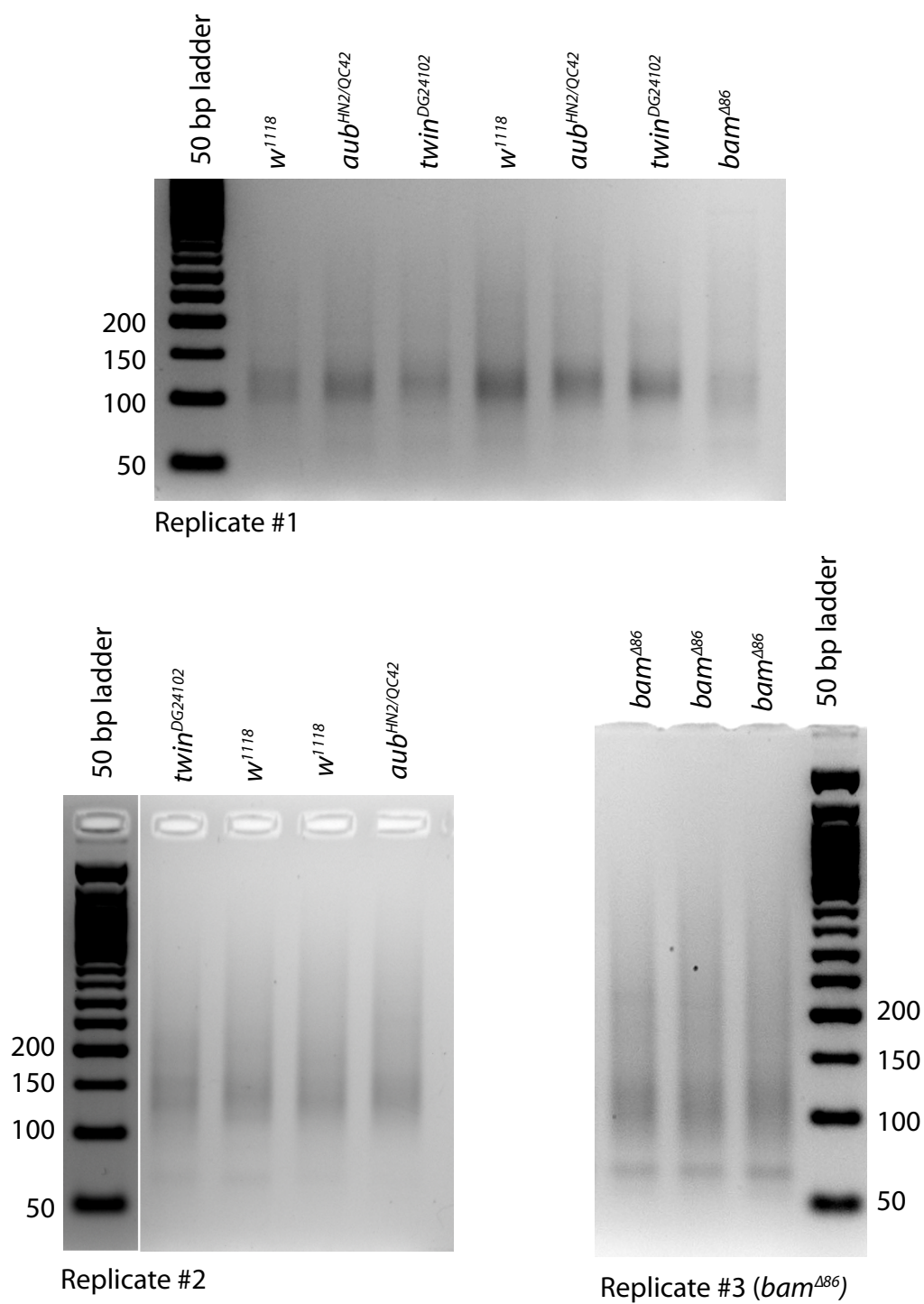

Source Data For Figure 4G

Supplement: Supplementary file 5 — Source Data for Figure 4G [file EMBJ-36-3194-s004.pdf]

WB: anti-Cbl (8C4)

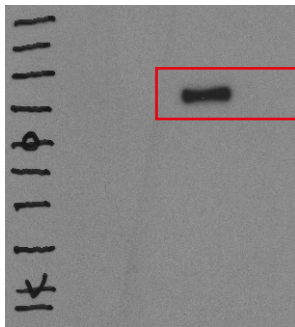

WB: anti-Cbl (10F1)

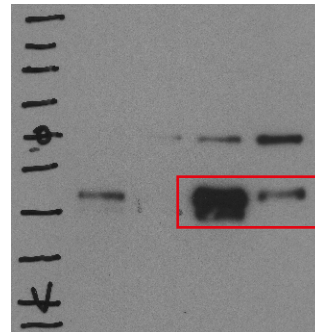

WB: anti-Actin

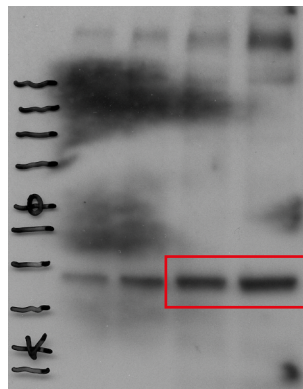

WB: anti-Actin

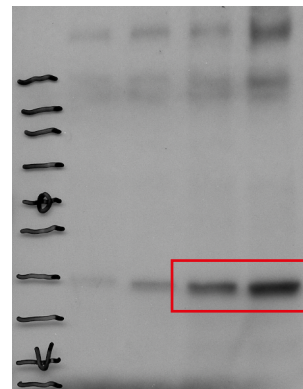

Source Data For Figure 6F

Supplement: Supplementary file 6 — Source Data for Figure 6F [file EMBJ-36-3194-s005.pdf]
